# Interpretable Multi-Head Self-Attention Architecture for Sarcasm Detection in Social Media

**DOI:** 10.3390/e23040394

**Published:** 2021-03-26

**Authors:** Ramya Akula, Ivan Garibay

**Affiliations:** Complex Adaptive Systems Lab, Department of Computer Science, University of Central Florida, Orlando, FL 32816, USA

**Keywords:** sarcasm detection, self-attention, interpretability, social media analysis

## Abstract

With the online presence of more than half the world population, social media plays a very important role in the lives of individuals as well as businesses alike. Social media enables businesses to advertise their products, build brand value, and reach out to their customers. To leverage these social media platforms, it is important for businesses to process customer feedback in the form of posts and tweets. Sentiment analysis is the process of identifying the emotion, either positive, negative or neutral, associated with these social media texts. The presence of sarcasm in texts is the main hindrance in the performance of sentiment analysis. Sarcasm is a linguistic expression often used to communicate the opposite of what is said, usually something that is very unpleasant, with an intention to insult or ridicule. Inherent ambiguity in sarcastic expressions make sarcasm detection very difficult. In this work, we focus on detecting sarcasm in textual conversations from various social networking platforms and online media. To this end, we develop an interpretable deep learning model using multi-head self-attention and gated recurrent units. The multi-head self-attention module aids in identifying crucial sarcastic cue-words from the input, and the recurrent units learn long-range dependencies between these cue-words to better classify the input text. We show the effectiveness of our approach by achieving state-of-the-art results on multiple datasets from social networking platforms and online media. Models trained using our proposed approach are easily interpretable and enable identifying sarcastic cues in the input text which contribute to the final classification score. We visualize the learned attention weights on a few sample input texts to showcase the effectiveness and interpretability of our model.

## 1. Introduction

Sarcasm is a rhetorical way of expressing dislike or negative emotions using exaggerated language constructs. It is an assortment of mockery and false politeness to intensify hostility without explicitly doing so. In face-to-face conversation, sarcasm can be identified effortlessly using facial expressions, gestures, and tone of the speaker. However, recognizing sarcasm in textual communication is not a trivial task as none of these cues are readily available. With the explosion of internet usage, sarcasm detection in online communications from social networking platforms, discussion forums, and e-commerce websites has become crucial for opinion mining, sentiment analysis, and identifying cyberbullies—online trolls. The topic of sarcasm received great interest from Neuropsychology [1] to Linguistics [2], but developing computational models for automatic detection of sarcasm is still at its nascent phase. Earlier works on sarcasm detection on texts use lexical (content) and pragmatic (context) cues [3] such as interjections, punctuation, and sentimental shifts, which are major indicators of sarcasm [4]. In these works, the features are hand-crafted and cannot generalize in the presence of informal language and figurative slang that is widely used in online conversations.

With the advent of deep-learning, recent works [5,6,7,8,9], leverage neural networks to learn both lexical and contextual features, eliminating the need for hand-crafted features. In these works, word embeddings are incorporated to train deep convolutional, recurrent, or attention-based neural networks to achieve state-of-the-art results on multiple large-scale datasets. While deep learning-based approaches achieve impressive performance, they lack interpretability. In this work, we also focus on the interpretability of the model along with its high performance. The main contributions of our work are as follows:

- Propose a novel, interpretable model for sarcasm detection using self-attention.
- Achieve state-of-the-art results on diverse datasets and exhibit the effectiveness of our model with extensive experimentation and ablation studies.
- Exhibit the interpretability of our model by analyzing the learned attention maps.

This paper is organized as follows: In Section 2 and Section 3, we briefly mention the related works and describe our proposed multi-head self-attention architecture. Section 4 includes details on model implementation, experiments, datasets, and evaluation metrics. Performance and attention analysis of our model are described in Section 5 and Section 6, followed by the conclusion of this work.

## 2. Related Work

Sarcasm has been studied for many decades in social sciences, yet developing methods to automatically identify sarcasm in texts is a fairly new field of study. The state-of-the-art automated sarcasm detection models can be broadly segregated into content- and context-based models.

In content-based approaches, lexical and linguistic cues, syntactic patterns are used to train classifiers for sarcasm detection. Carvalho et al. [10], González-Ibánez et al. [11], use linguistic features such as interjections, emoticons, and quotation marks. Tsur et al. [12], Davidov et al. [13] use syntactic patterns and lexical cues associated with sarcasm. The use of positive utterance in a negative context is used as a reliable feature to detect sarcasm by Riloff et al. [14]. Linguistic features such as implicit and explicit context incongruity, are used by Joshi et al. [4]. In these works, only the input text is used to detect sarcasm without any context information.

Context-based approaches increased in popularity in the recent past with the emergence of various online social networking platforms. As texts from these websites are prone to grammatical errors and extensive usage of slang, using context information helps better identify sarcasm. Wallace et al. [15], Poria et al. [16] detected sarcasm using sentiment and emotional information from the input text as contextual information. While, Amir et al. [17], Hazarika et al. [18] use personality features of the user as context, Rajadesingan et al. [19], Zhang et al. [20] use historical posts of the user to incorporate sarcastic tendencies. We show that context information, when available, helps improve the performance of the model but is not essential for sarcasm detection.

Existing works by Wallace et al. [15], Ptáček et al. [21], Wang et al. [22], Joshi et al. [23], use handcrafted features such as Bag of Words (BoW), Parts of Speech (POS), and sentiment/emotions to train their classifiers. Other works by Liu et al. [9], Poria et al. [16], Amir et al. [17], Zhang et al. [20], Ghosh and Veale [24], Vaswani et al. [25] use deep-learning to learn meaningful features and classify them. The method that uses handcrafted features is easily interpretable but lacks in performance. On the other hand, deep learning-based methods achieve high performance but lack interpretability.

In our work, we propose a deep learning-based architecture for sarcasm detection, which leverages self-attention to enable the interpretability of the model while achieving state-of-the-art performance on various datasets.

## 3. Proposed Approach

**Figure 1:**
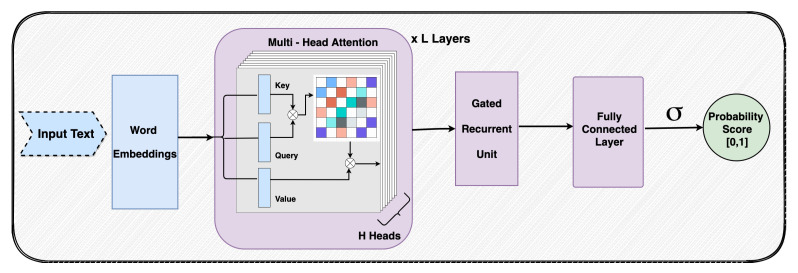
Multi-head self-attention architecture for sarcasm detection. Pre-trained word embeddings are extracted for input text and are enhanced by an attention module with *L* self-attention layers and *H* heads per layer. Resultant features are passed through a Gated Recurrent Unit and a Feed-Forward layer for classification.

Our proposed approach consists of five components: Data Pre-Processing, Multi-Head Self-Attention, Gated Recurrent Units (GRU), Classification, and Model Interpretability. The architecture of our sarcasm detection model is shown in Figure 1. Data pre-processing involves converting input text to word embeddings, which is required for training a deep learning model. To this end, we first apply a standard tokenizer (from [26]) to convert a sentence to a sequence of tokens, then we employ pre-trained language models to convert the tokens to word embeddings. These embeddings form the input to our multi-head self-attention module, which identifies words in the input text that provide crucial cues for sarcasm. In the next step, the GRU layer aids in learning long-distance relationships among these highlighted words and output a single feature vector that encodes the entire sequence. Finally, a fully-connected layer with sigmoid activation is used to obtain the final classification score.

### 3.1. Data Pre-Processing

Word embeddings range from the clustering of words based on the local context to the embeddings based on a global context that considers the association between a word and every other word in a sentence. Most popular ones that rely on local context are Continuous Bag of Words (CBOW), Skip Grams [27], and Word2Vec [28]. Other predictive models that capture global context are Global Vectors for word representation (GloVe) [29], FastText [30], Embeddings from Language Models (ELMO) [31] and Bidirectional Encoder Representations from Transformers (BERT) [32]. In our work, we employ word embedding that captures global context as we believe it is essential for detecting sarcasm. We show the results of the proposed approach using multiple word embeddings, including, BERT, ELMO, FastText, and GloVe.

### 3.2. Multi-Head Self-Attention

Given a sentence S, we apply a standard tokenizer and use pre-trained models to obtain *D* dimensional embeddings for individual words in the sentence. These embeddings *S* = {$e_{1}$, $e_{2}$, …, $e_{N}$}, $S\in R^{N\times D}$ from the input to our model. To detect sarcasm in sentence S, it is crucial to identify specific words that provide essential cues such as sarcastic connotations and negative emotions. The importance of these cue-words is dependent on multiple factors based on different contexts. In our proposed model, we leverage multi-head self-attention to identify these cue-words from the input text.

Attention is a mechanism to discover patterns in the input that are crucial for solving the given task. In deep learning, self-attention [25] is an attention mechanism for sequences, which helps learn the task-specific relationship between different elements of a given sequence to produce a better sequence representation. In the self-attention module, there are three linear projections: Key (*K*), Value (*V*), and Query (*Q*) of the given input sequence are generated, where $K,Q,V\in R^{N\times D}$. The attention map is computed based on the similarity between *K*, *Q*, and the output of this module $A\in R^{N\times D}$ is the scaled dot-product between *V* and the learned softmax attention ($QK^{T}$), as shown in Equation (1).

(1) $$A=\mathrm{softmax}\left(\frac{QK^{T}}{\sqrt{D}}\right)V$$

In multi-head self-attention, multiple copies of the self-attention module are used in parallel. Each head captures different relationships between the words in the input text and identifies those keywords that aid in classification. In our model, we use a series of multi-head self-attention layers (#*L*) with multiple heads (#*H*) in each layer.

### 3.3. Gated Recurrent Units

Self-attention finds the words in the text that are important in detecting sarcasm. These words can be close to each other or farther apart in the input text. To learn long-distance relationships between these words, we use GRUs. These units are an improvement over standard recurrent neural networks and are designed to dynamically remember and forget the information flow using Reset ($r_{t}$) and Update ($z_{t}$) gates to solve the vanishing gradient problem.

In our model, we use a single layer of bi-directional GRU to process the sequence *A*, as these units make use of the contextual information from both directions. Given the input sequence $A\in R^{N\times D}$, GRU computes hidden states *H* = {$h_{1}$, $h_{2}$, …, $h_{N}$}, $H\in R^{N\times D}$ for every element in the sequence as follows:

(2) $$\begin{array}{cc} r_{t} & =\sigma \left(W_{r}A_{t}+U_{r}h_{t-1}+b_{r}\right)\\ z_{t} & =\sigma \left(W_{z}A_{t}+U_{z}h_{t-1}+b_{z}\right)\\ {\tilde{h}}_{t} & =tanh\left(W_{h}A_{t}+U_{h}\left(r_{t}⊙h_{t-1}\right)+b_{h}\right)\\ h_{t} & =z_{t}⊙h_{t}+\left(1-z_{t}\right)⊙{\tilde{h}}_{t-1} \end{array}$$

where σ(.) is the element-wise sigmoid function and *W*, *U*, *b* are the trainable weights and biases. $r_{t}$, $z_{t}$, $h_{t}$, ${\tilde{h}}_{t}$∈$R^{d}$, where *d* is the size of the hidden dimension. We consider the final hidden state, $h_{N}$, which encodes all the information in the sequence, as an output from this module.

### 3.4. Classification

A single fully-connected feed-forward layer is used with sigmoid activation to compute the final output. Input to this layer is the feature vector $h_{N}$ from the GRU module and the output is a probability score y∈[0,1], computed as follows:

(3) $$y=\sigma \left(Wh_{N}+b\right),$$

where $W\in R^{d\times 1}$ are the weights of this layer and *b* is the bias term. Binary Cross Entropy (BCE) loss between the predicted output *y* and the ground-truth label $\hat{y}$ is used to train the model.

(4) $$loss\left(y,\hat{y}\right)=\hat{y}log\left(y\right)+\left(1-\hat{y}\right)log\left(1-y\right)$$

where $\hat{y}\in \left\{0,1\right\}$ is the binary label i.e., 1:Sarcasm and 0:No-sarcasm.

### 3.5. Model Interpretability

Developing models that can explain their predictions is crucial to building trust and faith in deep learning, while enabling a wide range of applications with machine intelligence at its backbone. Existing deep learning network architectures such as convolutional and recurrent neural networks are not inherently interpretable and require additional visualization techniques [33,34]. To avoid this, we employ inherently interpretable self-attention that allows the identification of elements in the input that are crucial for a given task.

## 4. Experiments

### 4.1. Datasets

Dataset details presented in Table 1, includes data source and the sample counts in train & test splits. These are sourced from varied online platforms including social networks and discussion forums.

#### 4.1.1. Twitter, 2013

In this dataset [14], the tweets that contain sarcasm are identified and labeled by the human annotators solely based on the contents of the tweets. These tweets do not depend on prior conversational context. Tweets with no sarcasm or those that required prior conversational context are labeled as non-sarcastic. As a pre-processing step, URLs are removed from the tweets and all mentions are replaced with @user.

#### 4.1.2. Dialogues, 2016

This Sarcasm Corpus V2 Dialogues dataset [35] is part of the Internet Argument Corpus [36], which includes annotated quote–response pairs for sarcasm detection. General sarcasm, hyperbole, and rhetorical are the three categories in this dataset. In these quote–response pairs, a quote is a dialogic parent to the response. Therefore, a response post can be mapped to the same quote post or the post earlier in the thread. Here, the quoted text is used as a context for sarcasm detection.

#### 4.1.3. Twitter, 2017

In this dataset [5], tweets are collected using a Twitter bot named *@onlinesarcasm*. This dataset not only contains tweets and replies to these tweets but also the mood of the user at the time of tweeting. The tweets/re-tweets of the users are the content and the replies to the tweets are the context. Similar to Twitter 2013 dataset, tweets in this dataset are pre-processed by removing URLs and replacing mentions.

#### 4.1.4. Reddit, 2018

Self-annotated corpus for sarcasm, SARC 2.0 dataset [37] contains comments from Reddit forums. Sarcastic comments by users are scrapped that are self-annotated by them using an \s token to indicate sarcastic intent. In our experiments, we use only the original comment without using any parent or child comments. “Main Balanced” and “Political” variants of the dataset are used in our experiments, the latter consists of comments only from the political subreddit.

#### 4.1.5. Headlines, 2019

This news headlines dataset [38] is collected from two news websites: the Onion and Huffpost. The Onion has sarcastic versions of current events, whereas Huffpost has real news headlines. Headlines are used as content and the news article is used as context.

### 4.2. Implementation Details

We implement our model in PyTorch [39], a deep-learning framework in Python. To tokenize and extract word embeddings for the input text, we use publicly available resources [26]. Specifically, we use tokenizer and pre-trained weights from the “bert-base-uncased” model to convert words to tokens and then convert tokens to word embeddings. The pre-trained BERT model is trained with inputs of maximum length, N=512 by truncating longer inputs and padding shorter inputs with special token <pad>. To extract the word embeddings, the weights of this pre-trained BERT model are frozen and inputs are truncated or padded (with token <pad>) based on their length. We consider the 768-dimensional output, for each word in the input, from the final hidden layer of the BERT model as the word embeddings. These embeddings for the words in the input text are passed through a series of multi-head self-attention layers #L, with multiple heads #H in each of the layers. The output from the self-attention layer is passed through a single bi-directional GRU layer with its hidden dimension d=512. The 512-dimensional output feature vector from the GRU layer is passed through the fully connected layer to yield a 1-dimensional output. A sigmoid activation is applied to the final output and BCE loss is used to compute the loss between the ground truth and the predicted probability score. The parameters in our model include weights from the Multi-Head Attention, GRU, and Fully Connected layers. When using the BERT model for extracting word embeddings, we initialize it with pre-trained weights and freeze them while training our model. We use the Adam optimizer to train our model with approximately 13 million parameters, using a learning rate of 1e-4, batch size of 64, and dropout set of 0.2. We use one NVIDIA Pascal Titan-X with 16 GB of memory for all our experiments. We set #H = 8 and #L = 3 in all our experiments for all the datasets.

### 4.3. Evaluation Metrics

We pose Sarcasm Detection as a classification problem and use Precision, Recall, F1-Score, and Accuracy as evaluation metrics to test the performance of the trained models. *Precision:* Ratio of the number of correctly predicted sarcastic sentences to the total number of predicted sarcastic sentences. *Recall:* Ratio of correctly predicted sarcastic sentences to the actual number of sarcastic sentences in the ground-truth. *F-score:* Harmonic mean of precision and recall. We use a threshold of 0.5 on the predictions from the model to compute these scores. Apart from these standard metrics, we also compute the Area Under the ROC Curve (AUC score), which is threshold independent.

## 5. Results

In this section, we present the results of our experiments on multiple publicly available datasets. The results on Twitter datasets are presented in Table 2 and Table 3. In the experiments with the Ghosh and Veale [5] dataset, we do not use any additional information about the user or the context tweets. Hence, for a fair comparison, we present the results on this dataset under the TTEA (Target Tweet Excluding Addressee) configuration. As evident from these tables, our multi-head self-attention model outperforms previous methods by a considerable margin. In Table 4, we present the results on the Reddit SARC 2.0 dataset, which is divided into two subsets: Main and Political. In both datasets, our proposed approach outperforms previous methods.

Apart from Twitter and Reddit data, we also experimented with data from other data sources such as Political Dialogues [35] and News Headlines [38]. In Table 5, we present the results on the Sarcasm Corpus V2 Dialogues dataset and in Table 6, we present the results on the News Headlines dataset. In both datasets, we see considerable improvements.

### 5.1. Ablation Study

The Sarcasm Corpus V2 Dialogues dataset [35] is used for the following ablations.

#### 5.1.1. Ablation 1

We vary the number of self-attention layers and fix the number of heads per layer (#H = 8). From the results of this experiment presented in Table 7, we observe that as the number of self-attention layers increases (#L = 0, 1, 3, 5), the improvement in the performance of the model due to the additional layers becomes saturated. Due to memory constraints, it is not feasible to have more than five self-attention layers in the model. However, these results show that the proposed multi-head self-attention model achieves a 2% improvement over the baseline model where only a single GRU layer is used without any self-attention layers.

#### 5.1.2. Ablation 2

We vary the number of heads per layer with a fixed number of self-attention layers (#L = 3). The results of these experiments are presented in Table 8. We observe that the performance of the model also increases with the increase in the number of heads per self-attention layer.

#### 5.1.3. Ablation 3

To further show the strength of our proposed network architecture, we perform this ablation, in which we train our model with different word embedding such as Glove-6B, Glove-840B, ELMO, and FastText and present the results in Table 9. These results show that the performance of our model is not due to the choice of word embeddings. With #H = 8 and #L = 3, the maximum possible batch size to train the model on 1 GPU with 16 GB memory is 64. We set #H = 8 and #L = 3 in all our experiments for all the datasets.

## 6. Model Interpretability

**Figure 2:**
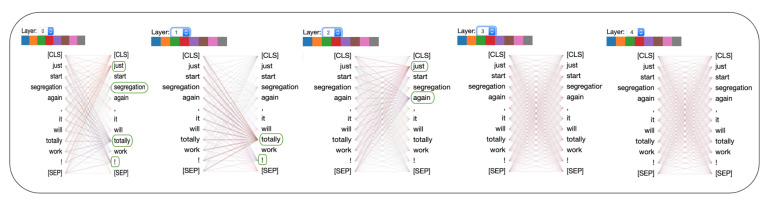
Attention analysis with sample sentence with sarcasm. Words providing cues for sarcasm, highlighted in green, are the words with higher attention weights. The prediction score for this sentence by our model is 0.94.

**Figure 3:**
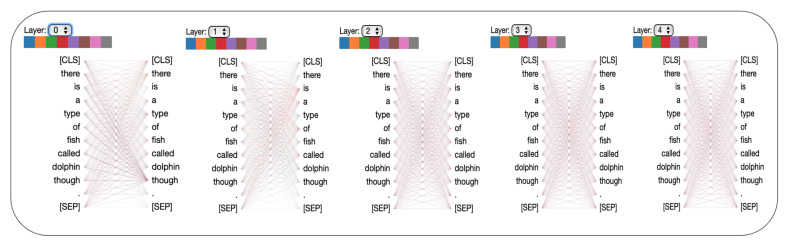
Attention analysis with sample sentence without sarcasm. Due to no presence of cues for sarcasm, every word in a sentence has a similar attention weight. The prediction score for this sentence by our model is 0.15.

**Figure 4:**
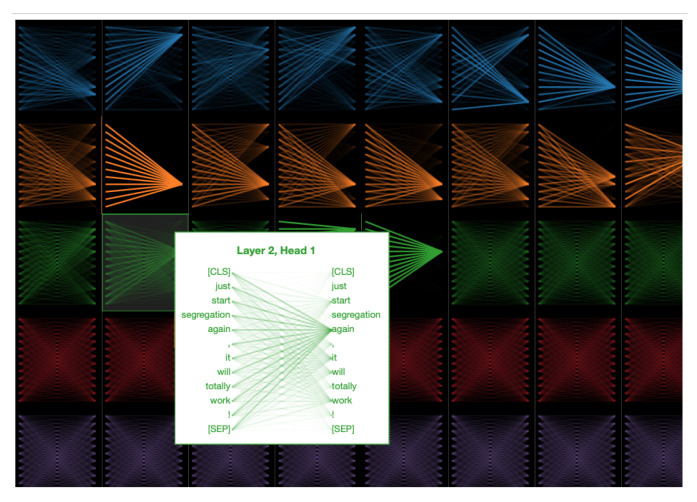
Attention analysis with sample sentence with sarcasm. Rows correspond to the different layers in the model and the columns correspond to the individual heads with a layer. When the input sentence contains sarcasm, we observe multiple heads, across layers attending to cue words in the input.

**Figure 5:**
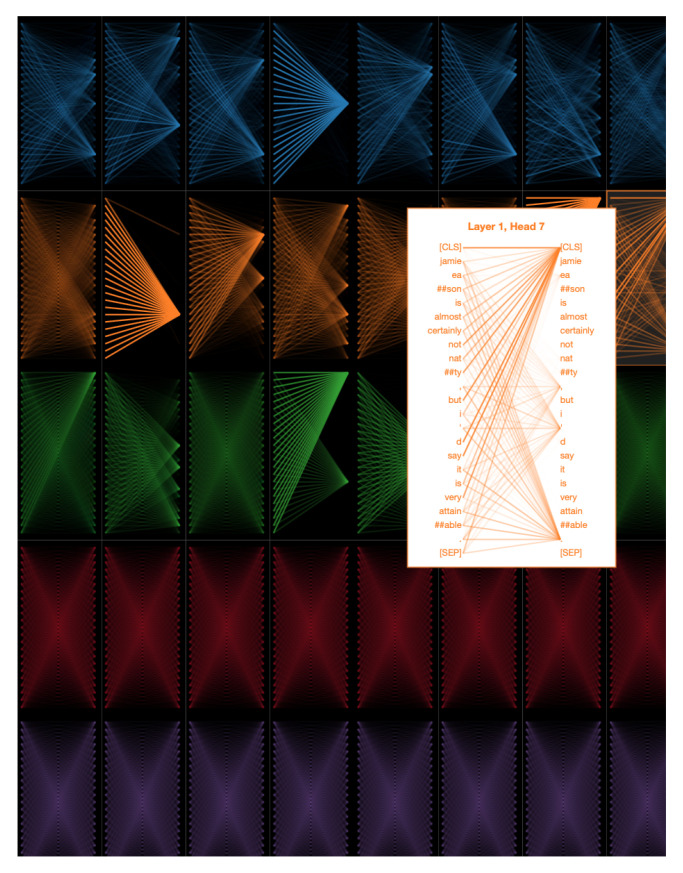
Attention analysis with sample sentence without sarcasm. Rows correspond to the different layers in the model and the columns correspond to the individual heads with a layer. When the input sentence contains no sarcasm, we observe that attention is distributed between multiple words in each head, across layers.

Attention maps from the individual heads of the self-attention layers provide the learned attention weights for each time-step in the input. In our case, each time-step is a word and we visualize the per-word attention weights for sample sentences with and without sarcasm from the SARC 2.0 Main dataset. The model we used for this analysis has five attention layers with eight heads per attention. Figure 2 and Figure 3 show attention analysis [42] for two sample sentences with and without sarcasm, respectively. Each column in these figures corresponds to a single attention layer and attention weights between words in each head are represented using colored edges. The darkness of an edge indicates the strength of the attention weight. CLS and SEP are classification and separator tokens from BERT. Figure 4 and Figure 5 are yet another visualization that provides a birds-eye view of attention across all the heads and layers in the model. Here rows correspond to five attention layers and the columns correspond to eight heads in each layer. From both the visualizations, we observe that words receiving the most attention vary between different heads in each layer and also across layers.

### 6.1. Attention Analysis

**Figure 6:**
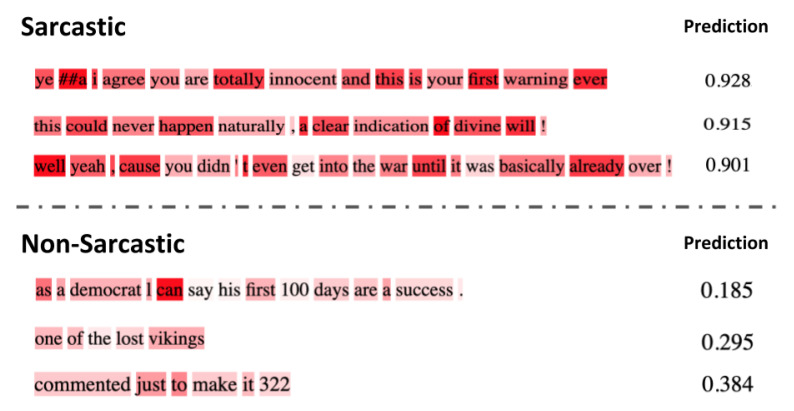
Visualization of the attention on individual words of sample sentences from both Sarcastic and Non-Sarcastic classes are shown in the column to the left. Probability scores predicted by our model are shown in the column to the right. High scores are predicted for sarcastic sentences and low scores for non-sarcastic sentences.

For a sentence with sarcasm, Figure 2 shows that certain words receive more attention than others. For instance, words such as ‘just’, ‘again’, ‘totally’, ‘!’, have darker edges connecting them with every other word in a sentence. These are the words in the sentence that hint at sarcasm and, as expected, these receive higher attention than others. Note that each cue word is attended by a different head in the first three layers of self-attention. In the final two layers, we observe that the attention is spread out to every word in the sentence, indicating redundancy of these layers in the model. A sample sentence shown in Figure 3 has no sarcasm, thus no word is highlighted by any head in any layer. In Figure 6, we visualize the distribution of attention over the words in a sentence for six sample sentences. Attention weight for a word is computed by first considering the maximum attention it receives across layers and then averaging the weights across multiple-heads in the layer. Finally, the weights for a word are averaged over all the words in the sentence. The stronger the highlight for a word, the higher the attention weight placed on it by the model while classifying the sentence. Words from the sarcastic sentences with higher weights show that the model can detect sarcastic cues from the sentence. For example, the words “totally”, “first”, “ever” from the first sentence and “even”, “until”, “already” from the third sentence. These are the words that exhibit sarcasm in the sentences, which the model can successfully identify. In all the samples that are classified as non-sarcasm, the weights for the individual words are very low in comparison to cue-words from the sarcastic sentences. The probability of sarcasm predicted by our model for each of the sentences is shown on the right and their respective scores on the left column in Figure 6. Our model can predict a high score for sarcastic sentences and low scores for non-sarcastic sentences.

### 6.2. Failure Cases

**Figure 7:**
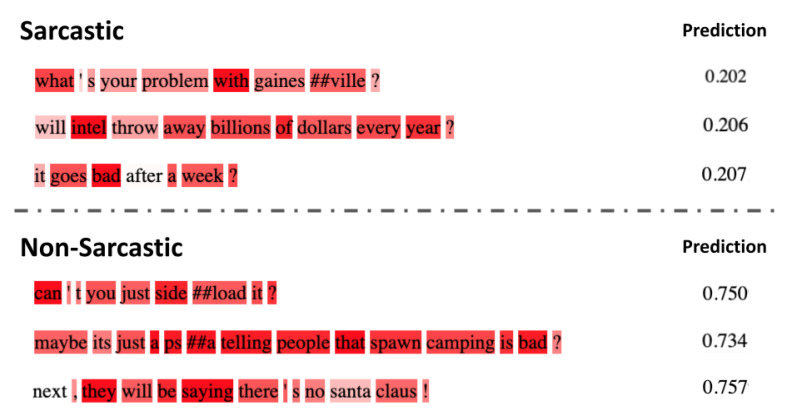
Sample sentences that our model fails to classify correctly. The top row shows Sarcastic sentences with predicted probability of Sarcasm less than 0.5 and the bottom row shows a Non-Sarcastic sentence with probability greater than 0.5. It can be observed from these examples that our model has difficulty in detecting sarcasm when the inputs sentences are questions.

In this section, we provide a brief analysis of the failure cases. We present a few samples that our model fails to classify correctly in Figure 7. From the analysis of such failure cases, we observe that our model mostly finds it difficult to classify interrogative sentences which usually end with a “?”. With no context information, we believe classifying these correctly is a challenging task not only to the deep learning models but also to human annotators.

Apart from these interrogative sentences, we also show a sample Non-Sarcastic sentence that our model classifies incorrectly as Sarcastic. For example, if we observe the third sample in the Non-Sarcastic part of the Figure 7; here the sample sentence ends with an exclamation “!”, illustrating hard sample to classify correctly without prior knowledge.

## 7. Conclusions

In this work, we propose a novel multi-head self-attention-based neural network architecture to detect sarcasm in a given sentence. Our proposed approach has five components: data pre-processing, multi-head self-attention module, gated recurrent unit module, classification, and model interpretability. Multi-head self-attention is used to highlight the parts of the sentence that provide crucial cues for sarcasm detection. GRUs aid in learning long-distance relationships among these highlighted words in the sentence. The output from this layer is passed through a fully-connected classification layer to obtain the final classification score. The experiments were conducted on multiple datasets from varied data sources and show significant improvement over the state-of-the-art models by all evaluation metrics. The results from ablation studies and analysis of the trained model are presented to show the importance of different components of our model. We analyze the learned attention weights to interpret our trained model and show that it can indeed identify words in the input text that provide cues for sarcasm.

## Figures and Tables

**Table 1:** Statistics of datasets used in our experiments. Twitter, 2013 [14], Dialogues, 2016 [35], Twitter, 2017 [5], Reddit, 2018 [37], and Headlines, 2019 [38].

Source	Train	Test	Total	Sarcastic	Non Sarcastic
Twitter, 2013	1368	588	1956	308	1648
Dialogues, 2016	3754	938	4692	2346	2346
Twitter, 2017	51,189	3742	54,931	25,872	29,059
Reddit, 2018	154,702	64,666	219,368	109,684	109,684
Headlines, 2019	22,895	5724	28,619	13,634	14,985

**Table 2:** Results on Twitter dataset [14].

Models	Precision	Recall	F1	AUC
NBOW	71.2	62.3	64.1	-
Vanilla CNN	71.0	67.1	68.5	-
Vanilla LSTM	67.3	67.2	67.2	-
Attention LSTM	68.7	68.6	68.7	-
Bootstrapping [14]	62.0	44.0	51.0	-
EmotIDM [40]	-	-	75.0	-
Fracking Sarcasm [24]	88.3	87.9	88.1	-
GRNN [20]	66.3	64.7	65.4	-
ELMo-BiLSTM [6]	75.9	75.0	75.9	-
ELMo-BiLSTM FULL [6]	77.8	73.5	75.3	-
ELMo-BiLSTM AUG [6]	68.4	70.8	69.4	-
A2Text-Net [9]	91.7	91.0	90.0	97.0
**Our Model**	**97.9**	**99.6**	**98.7**	**99.6**
	(+6.2 ↑)	(+8.6 ↑)	(+8.7 ↑)	(+2.6 ↑)

**Table 3:** Results on Twitter dataset [5].

Models	Precision	Recall	F1	AUC
Sarcasm Magnet [5]	73.3	71.7	72.5	-
Sentence-level attention [7]	74.9	75.0	74.9	-
Self Matching Networks [8]	76.3	72.5	74.4	-
A2Text-Net [9]	80.3	80.2	80.1	88.4
**Our Model**	**80.9**	**81.8**	**81.2**	**88.6**
	(+0.6 ↑)	(+1.6 ↑)	(+1.1 ↑)	(+0.2 ↑)

**Table 4:** Results on Reddit dataset SARC 2.0 and SARC 2.0 Political [37].

Models	Main-Balanced		Political	
	Accuracy	F1	Accuracy	F1
Bag-of-words	63.0	64.0	59.0	60.0
CNN	65.0	66.0	62.0	63.0
CNN-SVM [16]	68.0	68.0	70.65	67.0
CUE-CNN [17]	70.0	69.0	69.0	70.0
CASCADE [18]	77.0	77.0	74.0	75.0
SARC 2.0 [37]	75.0	-	76.0	-
ELMo-BiLSTM [6]	72.0	-	78.0	-
ELMo-BiLSTM FULL [6]	76.0	76.0	72.0	72.0
**Our Model**	**81.0**	**81.0**	**80.0**	**80.0**
	(+4.0 ↑)	(+4.0 ↑)	(+2.0 ↑)	(+5.0 ↑)

**Table 5:** Results on Sarcasm Corpus V2 Dialogues dataset [35].

Models	Precision	Recall	F1	AUC
NBOW	66.0	66.0	66.0	-
Vanilla CNN	68.4	68.1	68.2	-
Vanilla LSTM	68.3	63.9	60.7	-
Attention LSTM	70.0	69.6	69.6	-
GRNN [20]	62.2	61.8	61.2	-
CNN-LSTM-DNN [24]	66.1	66.7	65.7	-
SIARN [41]	72.1	71.8	71.8	-
MIARN [41]	72.9	72.9	72.7	-
ELMo-BiLSTM [6]	74.8	74.7	74.7	-
ELMo-BiLSTM FULL [6]	76.0	76.0	76.0	-
**Our Model**	**77.4**	**77.2**	**77.2**	**0.834**
	(+1.2 ↑)	(+1.4 ↑)	(+1.2 ↑)	

**Table 6:** Results on New Headlines dataset [38].

Models	Precision	Recall	F1	Accuracy	AUC
Hybrid [38]	-	-	-	89.7	-
A2Text-Net [9]	86.3	86.2	86.2	-	0.937
**Our Model**	**0.919**	**91.8**	**91.8**	**91.6**	**97.4**
	(+5.6 ↑)	(+5.6 ↑)	(+5.6 ↑)	(+1.9 ↑)	(+3.7 ↑)

**Table 7:** Ablation study with a varying number of attention layers #*L* and fixed Heads **#*H* = 8** on the Sarcasm Corpus V2 Dialogues dataset [35].

#*L*-Layers	Precision	Recall	F1
0 (GRU only)	75.6	75.6	75.6
1 Layer	76.2	76.1	76.1
3 Layers	77.4	77.2	77.2
5 Layers	77.6	77.6	77.6

**Table 8:** Ablation study with varying number of Heads #*H* and fixed Layers **#*L* = 3** on the Sarcasm Corpus V2 Dialogues dataset [35].

#*H*-Heads	Precision	Recall	F1
1 Head	74.9	74.5	74.4
4 Heads	76.9	76.8	76.8
8 Heads	77.4	77.2	77.2

**Table 9:** Ablation study on various word embeddings on the Sarcasm Corpus V2 Dialogues dataset [35].

Models	Embeddings	Precision	Recall	F1	AUC
MIARN [41]	-	72.9	72.9	72.7	-
ELMo-BiLSTM FULL [6]	ELMO	76.0	76.0	76.0	-
Our Model	BERT	77.4	77.2	77.2	83.4
	ELMO	76.7	76.7	76.7	80.8
	FastText	75.7	75.7	75.7	81.6
	Glove 6B	76.0	76.0	76.0	82.3
	Glove 840B	77.0	77.0	77.0	82.9

## Data Availability

In this work, we employ publicly available datasets. Required information on these datasets are provided in Section 4.1.
